# Switching patients with acromegaly from octreotide to pasireotide improves biochemical control: crossover extension to a randomized, double-blind, Phase III study

**DOI:** 10.1186/s12902-016-0096-8

**Published:** 2016-04-02

**Authors:** Marcello D. Bronstein, Maria Fleseriu, Sebastian Neggers, Annamaria Colao, Michael Sheppard, Feng Gu, Chiung-Chyi Shen, Mônica Gadelha, Andrew J. Farrall, Karina Hermosillo Reséndiz, Matthieu Ruffin, YinMiao Chen, Pamela Freda, on behalf of the Pasireotide C2305 Study Group

**Affiliations:** Neuroendocrine Unit, Division of Endocrinology and Metabolism, University of São Paulo Medical School, São Paulo, Brazil; Department of Medicine and Neurological Surgery, Northwest Pituitary Center, Oregon Health & Science University, Portland, OR USA; Department of Medicine, Erasmus University Medical Center, Rotterdam, The Netherlands; Dipartimento di Medicina Clinica e Chirurgia, Università Federico II di Napoli, Naples, Italy; Centre for Endocrinology, Diabetes and Metabolism, University of Birmingham, Edgbaston, Birmingham, UK; Department of Endocrinology, Key Laboratory of Endocrinology, Ministry of Health, Peking Union Medical College Hospital, Beijing, China; Department of Minimally Invasive Skull Neurosurgery, Neurological Institute, Taichung Veterans General Hospital, Taichung, Taiwan; Graduate Institute of Medical Sciences, National Defense Medical Center, Taipei, Taiwan; Department of Physical Therapy, Hungkuang University, Taichung, Taiwan; Endocrine Unit, Hospital Universitário Clementino Fraga Filho, Universidade Federal do Rio de Janeiro, Rio de Janeiro, Brazil; Brain Research Imaging Centre, University of Edinburgh, Edinburgh, UK; Clinical Development, Novartis Pharmaceuticals Corporation, Florham Park, NJ USA; Clinical Development, Oncology Business Unit, Novartis Pharma AG, Basel, Switzerland; Department of Medicine, Columbia University College of Physicians & Surgeons, William Black Medical Res. Building, Room 9-905, 650 W. 168th Street, New York, NY 10032 USA

**Keywords:** Pasireotide, Octreotide, Acromegaly, Extension, Crossover

## Abstract

**Background:**

Many patients with acromegaly do not achieve biochemical control with first-generation somatostatin analogues. A large, multicenter, randomized, Phase III core study demonstrated that pasireotide LAR had significantly superior efficacy over octreotide LAR. This analysis explores the efficacy and safety of switching therapeutic arms in inadequately controlled patients during a 12-month crossover extension.

**Methods:**

Patients with inadequate biochemical control (GH ≥2.5 μg/L and/or IGF-1 > ULN) at end of core study (month 12) were eligible to switch to pasireotide LAR 40 mg/28 days (*n* = 81) or octreotide LAR 20 mg/28 days (*n* = 38). One dose escalation to pasireotide LAR 60 mg/28 days or octreotide LAR 30 mg/28 days was permitted, but not mandatory, at month 17 or 20.

**Results:**

Twelve months after crossover, 17.3 % of pasireotide LAR and 0 % of octreotide LAR patients achieved GH <2.5 μg/L and normal IGF-1 (main outcome measure); 27.2 and 5.3 % of pasireotide LAR and octreotide LAR patients achieved normal IGF-1, respectively; 44.4 and 23.7 % of pasireotide LAR and octreotide LAR patients achieved GH <2.5 μg/L, respectively. Mean (±SD) tumor volume further decreased from the end of the core study by 25 % (±25) and 18 % (±28); 54.3 % of pasireotide LAR and 42.3 % of octreotide LAR patients achieved significant (≥20 %) tumor volume reduction during the extension. The safety profile of pasireotide LAR was similar to that of octreotide LAR, with the exception of the frequency and degree of hyperglycemia-related adverse events.

**Conclusions:**

Pasireotide LAR is a promising treatment option for patients with acromegaly inadequately controlled with the first-generation somatostatin analogue octreotide LAR.

**Trial registration:**

clinicaltrials.gov, NCT00600886. Registered 14 January 2008

**Electronic supplementary material:**

The online version of this article (doi:10.1186/s12902-016-0096-8) contains supplementary material, which is available to authorized users.

## Background

Acromegaly is a rare disorder most commonly caused by prolonged secretion of excess growth hormone (GH) from a pituitary adenoma. In the absence of adequate treatment, acromegaly is associated with increased morbidity and mortality [1, 2]. The goals of therapy are to control the levels of both GH and insulin-like growth factor 1 (IGF-1), reduce and/or stabilize tumor size, preserve pituitary function and prevent recurrence. A recent consensus statement on the management of acromegaly advocates reducing GH and IGF-1 levels to as close to normal as possible (GH <1.0 μg/L [using an ultrasensitive assay] and IGF-1 within the normal range for age and sex) [3, 4]. Earlier studies measuring GH by radioimmunoassay indicated that suppression of random GH to <2.5 μg/L and normalization of IGF-1 levels are associated with restoration of mortality to that of a standard population [5].

Somatostatin analogues are the mainstay of medical therapy for patients with acromegaly who have not achieved biochemical control after transsphenoidal surgery. They can also be used as first-line treatment when the chance of surgical cure is low (invasive macroadenomas), when surgery is contraindicated, or for patients who refuse surgery [6]. However, many patients do not achieve biochemical control with first-generation somatostatin analogues [7–12]. In those patients who do not fully respond to somatostatin analogue monotherapy, recent clinical practice guidelines have advocated the addition of a dopamine agonist (cabergoline) or a GH receptor antagonist (pegvisomant) to somatostatin analogue treatment [13].

Pasireotide, a multireceptor-targeted somatostatin analogue, was rationally designed with a broader somatostatin receptor (SSTR) binding profile than first-generation somatostatin analogues to achieve higher response rates. In a large, randomized, double-blind, Phase III trial (core phase of this study), pasireotide long-acting release (LAR) was significantly superior to octreotide LAR at providing GH <2.5 μg/L and normal IGF-1 at month 12 in medically naïve patients with acromegaly [14]. The extension phase described here allowed patients with inadequate biochemical control at month 12 to switch treatment at month 13 (ie from octreotide LAR to pasireotide LAR or vice versa). This manuscript reports the efficacy and safety results at the end of the crossover extension phase.

## Methods

### Extension study: patients could cross over or continue

This was a planned double-blind extension study to a 12-month, randomized, double-blind, multicenter, Phase III trial (the core study). In the core study, patients were randomized to pasireotide LAR 40 mg every 28 days or octreotide LAR 20 mg every 28 days. Titration to pasireotide LAR 60 mg or octreotide LAR 30 mg at month 3 or 7 was permitted, but not mandatory, based on biochemical response. Dose decreases (pasireotide LAR 60 to 40 mg and 40 to 20 mg; octreotide LAR 30 to 20 mg and 20 to 10 mg) were permitted for tolerability, as was an increase to the original dose upon resolution. Patients who completed month 12 of the core study could enroll either to continue receiving their randomized therapy or to switch therapy, depending on biochemical response, at the end of the core study:

- Patients with GH ≥2.5 μg/L and/or IGF-1 > ULN (upper limit of normal; age and sex matched) could switch treatment to either pasireotide LAR 40 mg every 28 days or octreotide LAR 20 mg every 28 days at month 13. After crossover, patients could receive octreotide LAR for 12 months or pasireotide LAR for as long as clinical benefit was obtained. Among patients who crossed over, one dose escalation to pasireotide LAR 60 mg every 28 days or octreotide LAR 30 mg every 28 days was permitted, but not mandatory, at month 17 or 20. The results from these patients are reported in this manuscript.
- Patients with biochemical control (GH <2.5 μg/L and IGF-1 ≤ ULN) at month 12 could continue receiving their randomized therapy. At the discretion of the investigator, patients without biochemical control but with clinical benefit derived from the study drug could receive their randomized therapy during the extension. Results from these patients are reported in a separate manuscript [15].

The study was conducted in accordance with the Declaration of Helsinki, and an independent ethics committee or institutional review board for each study site approved the study protocol. See Additional file 1 for further details. All patients provided written informed consent to participate in the trial (trial identifier: NCT00600886).

### Crossover phase

#### Patients and endpoints

The core study enrolled adult patients with active acromegaly (GH >5 μg/L or lack of suppression of GH nadir to <1 μg/L after oral glucose tolerance test, and IGF-1 above the age-adjusted ULN) who had never received medical therapy for acromegaly. Patients were eligible to enroll if they had undergone ≥1 pituitary surgery, or if they were *de novo* because of refused pituitary surgery or contraindication for surgery; patients were not eligible to participate if they had undergone pituitary irradiation within the last 10 years. The full entry criteria have been described previously [14].

#### Main endpoints

The main endpoint of the crossover phase was the proportion of patients with both GH <2.5 μg/L and normal IGF-1 for age and sex 12 months after switching medical therapy because of inadequate biochemical control (GH ≥2.5 μg/L and/or IGF-1 > ULN). GH levels were determined by a 2-h five-point mean on the morning of study-drug injection. IGF-1 sampling was performed immediately before study-drug injection. See the Additional file 1 for further details.

#### Additional endpoints

Other endpoints included the proportion of patients achieving GH <2.5 μg/L, the proportion of patients achieving normal IGF-1, changes from extension baseline (defined as last assessment prior to crossover) in GH and IGF-1, changes from extension baseline in tumor volume, changes from extension baseline in signs and symptoms, and safety after switching therapy. Gadolinium-enhanced pituitary magnetic resonance imaging was performed at extension baseline and 12 months after crossover and evaluated by a central reader blinded to treatment. A pituitary tumor volume change of ≥20 % from extension baseline was considered significant. Tumor volume was calculated by hand drawing around the tumor circumference in coronal cross-sections, multiplying the area by slice thickness, and summing the resulting volumes across all slices containing tumor. See the Additional file 1 for further details.

Five symptoms of acromegaly (headache, fatigue, perspiration, paresthesia and osteoarthralgia) were scored from 0 (no symptom) to 4 (very severe). Health-related quality of life (HRQoL) was assessed each month using the AcroQoL questionnaire, which is a 22-item instrument that results in scores ranging from 0 (worst HRQoL) to 100 (best HRQoL) [16, 17].

Safety was assessed according to the National Cancer Institute Common Terminology Criteria for Adverse Events (CTCAE) version 3.0 [18] and consisted of: monitoring and recording of all adverse events (AEs); regular monitoring of hematology, blood chemistry and urinalysis parameters; performance of physical examinations; and body weight measurements. Blood samples for laboratory tests, including blood glucose measurements, were drawn at each visit under fasted conditions before the morning dose of study drug. AEs experienced after switching treatments are reported and were classified as grade 1 (mild), 2 (moderate), 3 (severe), or 4 (life threatening or disabling).

#### End of study

The last assessment of GH, IGF-1 and tumor volume before the end of the planned extension phase was performed at month 25; therefore, efficacy data are reported up to month 25. Safety data are presented for all patients after crossover until month 26.

#### Statistical analyses

Descriptive summary statistics were provided for the crossover data. No formal statistical tests were planned to compare the treatment arms during the crossover phase. The extension was not designed to determine a difference in efficacy or safety outcomes between the two groups. Results include all patients with available data for a given measure. GH and IGF-1 samples were considered missing if they were taken >35 days after injection.

### Protocol amendment

**Fig. 1:**
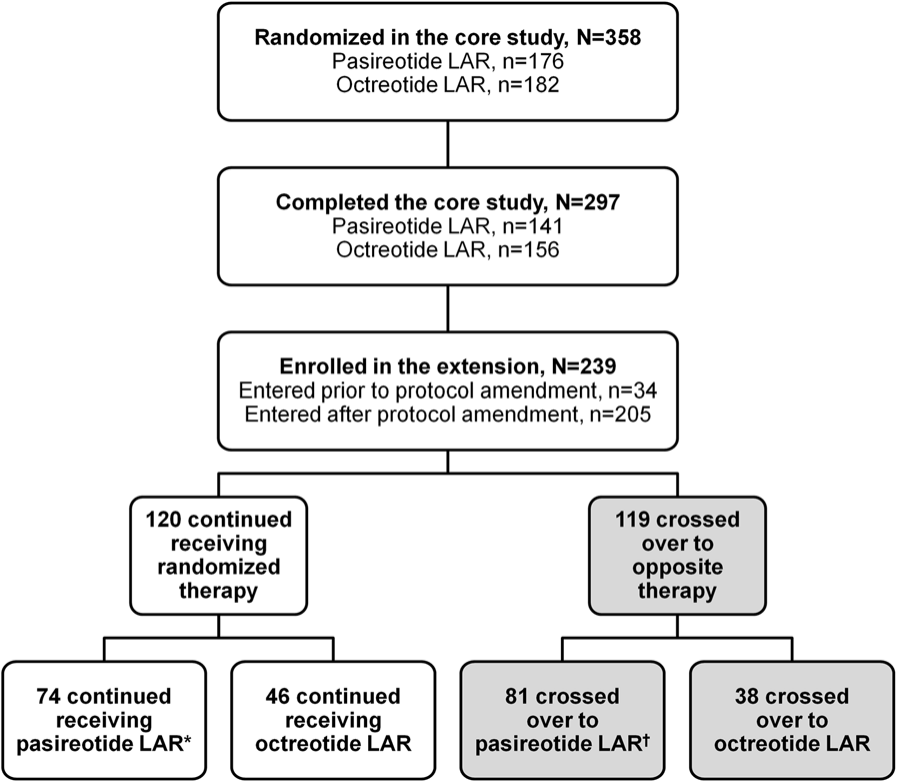
Patient disposition. Flowchart showing the number of patients who were randomized, completed the 12-month core study, entered the extension on their randomized treatment, and entered the extension after switching to the opposite treatment. Thirty-four patients entered the extension phase prior to implementation of the protocol amendment and received unblinded treatment during the extension; of these: *15 patients continued to receive their randomized treatment with pasireotide LAR and ^†^19 crossed over from octreotide LAR to pasireotide LAR during the extension

Prior to implementation of a protocol amendment, patients randomized to pasireotide LAR in the core study did not have the option to switch to octreotide LAR at month 13. Additionally, patients randomized to octreotide LAR in the core study who achieved GH <2.5 μg/L and IGF-1 ≤ ULN at month 12 could not continue receiving octreotide LAR during the extension phase; these patients were considered to have completed the study at month 12 and were discontinued. The protocol amendment allowed patients to receive octreotide LAR in the extension phase as either continued or crossover therapy. Patients who entered the extension phase after the protocol amendment received treatment in a double-blind manner (*n* = 205), while those who entered prior to the amendment received unblinded pasireotide treatment for the duration of the extension (*n* = 34). Thirty-four patients entered the extension phase prior to the protocol amendment. Of these patients, 15 continued to receive their randomized treatment with pasireotide LAR and 19 crossed over from octreotide LAR to pasireotide LAR during the extension; the latter 19 patients are included in this manuscript (Fig. 1).

Nineteen and 13 patients in the pasireotide LAR and octreotide LAR arms, respectively, completed the core study prior to the protocol amendment but did not enter the extension phase. Based on manual review of the response status for each patient at month 12, it is estimated that 14 patients in the pasireotide LAR arm would have potentially been eligible to cross over to octreotide LAR during the extension phase had they reached the end of the core study after implementation of the amendment; four patients in the octreotide LAR arm would have potentially been eligible to continue receiving octreotide LAR.

## Results

### Patient characteristics, disposition and exposure to medication

In total, 358 patients entered the core study, and 141 (80.1 %) and 156 (85.7 %) pasireotide LAR and octreotide LAR patients, respectively, completed 12 months of treatment. The results of the core study have been described elsewhere [14].

Of the 119 patients who switched therapy at month 13, 81 crossed over to pasireotide LAR and 38 crossed over to octreotide LAR (Fig. 1). During the 12-month extension, discontinuation rates were 38.3 % (*n* = 31/81) and 34.2 % (*n* = 13/38) with pasireotide LAR and octreotide LAR, respectively. Reasons for discontinuation in the pasireotide LAR and octreotide LAR arms up to month 12 of the crossover phase were AEs (12 and 1 patients, respectively), withdrawn consent (8 and 4 patients, respectively), unsatisfactory therapeutic effect (7 and 4), administrative problems (1 and 4), patient no longer requires study drug (2 and 0), and abnormal laboratory values (1 and 0). The median (range) duration of treatment after crossover was 388 days (28–432) and 364 days (85–399) with a median of 14 and 13 injections received for the pasireotide LAR and octreotide LAR groups, respectively. Previous surgery (prior to enrollment in the core study) had been performed in 43 % (*n* = 35/81) and 26 % (*n* = 10/38) of patients switched to pasireotide LAR and octreotide LAR, respectively (Table 1); all remaining patients had not received any prior treatment for acromegaly. None of the patients who entered the crossover extension phase had previously received pituitary irradiation.

**Table 1:** Demographics and characteristics at core study baseline of patients who switched treatments

Demographic variable	Crossed over to pasireotide LAR (*N* = 81)	Crossed over to octreotide LAR (*N* = 38)
Median age (range), years	45.0 (24–85)	48.5 (25–64)
Female, n (%)	38 (46.9)	22 (57.9)
Race, n (%)		
Caucasian	42 (51.9)	20 (52.6)
Black	1 (1.2)	1 (2.6)
Asian	26 (32.1)	8 (21.1)
Native American	3 (3.7)	1 (2.6)
Other	9 (11.1)	8 (21.1)
Median time since diagnosis prior to enrollment in the core study, months	7.1	4.2
Previous surgery, n (%)	35 (43.2)	10 (26.3)
Previous irradiation, n (%)	0 (0)	0 (0)
Median time since surgery prior to enrollment in the core study, months	6.6	10.3

At extension baseline, mean GH and IGF-1 levels were 5.9 μg/L and 1.9×ULN in patients who crossed over to pasireotide LAR, and 7.1 μg/L and 2.1×ULN in patients who crossed over to octreotide LAR. Some patients who crossed over had control of either IGF-1 or GH at the end of the core study. Among patients who crossed over to pasireotide LAR, 34 (42.0 %) had GH <2.5 μg/L but not normal IGF-1 after 12 months of octreotide LAR during the core study; four (4.9 %) had achieved normal IGF-1 but not GH <2.5 μg/L. One patient who crossed over to pasireotide LAR was biochemically controlled (GH <2.5 μg/L and normal IGF-1) at month 12 of the core study; this patient had elevated GH and IGF-1 levels 21 days after administration of the final dose of octreotide LAR during the core study. Among the patients who crossed over to octreotide LAR, eight (21.1 %) had GH <2.5 μg/L but not normal IGF-1 after 12 months of pasireotide LAR during the core study; two (5.3 %) had normal IGF­1 but not GH <2.5 μg/L. A total of 69 (85.2 %) and 34 (89.5 %) patients who crossed over to pasireotide LAR and octreotide LAR, respectively, had been uptitrated during the core study.

### Efficacy outcomes

#### Effect of treatment on GH and IGF-1 levels

Of the 81 patients inadequately controlled with octreotide LAR who switched to pasireotide LAR, 14 (17.3 %; 95 % CI: 9.8, 27.3) had biochemical control 12 months after crossover. A total of 36 patients (44.4 %) had GH <2.5 μg/L and 22 (27.2 %) had normal IGF-1 12 months after crossover. Of the 38 patients inadequately controlled with pasireotide LAR who switched to octreotide LAR, none had biochemical control 12 months after crossover. A total of nine patients (23.7 %) had GH <2.5 μg/L and two (5.3 %) had normal IGF-1 12 months after crossover (Table 2).

**Table 2:** Biochemical response rates at the end of the core study and after crossover, by treatment group

	Crossed over to pasireotide LAR (*N* = 81)		Crossed over to octreotide LAR (*N* = 38)	
	n (%)	95 % exact CI	n (%)	95 % exact CI
*GH <2.5 μg/L and normal IGF-1*				
End of core (month 12)	1 (1.2)	(0.0–6.7)	0 (0.0)	(0.0–9.3)
Month 3	14 (17.3)	(9.8, 27.3)	1 (2.6)	(0.1, 13.8)
Month 6	17 (21.0)	(12.7, 31.5)	1 (2.6)	(0.1, 13.8)
Month 9	18 (22.2)	(13.7, 32.8)	2 (5.3)	(0.6, 17.7)
Month 12	14 (17.3)	(9.8, 27.3)	0 (0.0)	–
*GH <2.5 μg/L*				
End of core (month 12)	35 (43.2)	(32.2–54.7)	8 (21.1)	(9.6–37.3)
Month 3	40 (49.4)	(38.1, 60.7)	11 (28.9)	(15.4, 45.9)
Month 6	35 (43.2)	(32.2, 54.7)	12 (31.6)	(17.5, 48.7)
Month 9	44 (54.3)	(42.9, 65.4)	12 (31.6)	(17.5, 48.7)
Month 12	36 (44.4)	(33.4, 55.9)	9 (23.7)	(11.4, 40.2)
*Normal IGF-1*				
End of core (month 12)	5 (6.2)	(2.0–13.8)	2 (5.3)	(0.6–17.7)
Month 3	16 (19.8)	(11.7, 30.1)	3 (7.9)	(1.7, 21.4)
Month 6	25 (30.9)	(21.1, 42.1)	3 (7.9)	(1.7, 21.4)
Month 9	24 (29.6)	(20.0, 40.8)	4 (10.5)	(2.9, 24.8)
Month 12	22 (27.2)	(17.9, 38.2)	2 (5.3)	(0.6, 17.7)

One patient who crossed over to pasireotide LAR had GH <2.5 μg/L *and* normal IGF-1 at month 12 of the core study; this patient had elevated GH and IGF-1 at last assessment prior to the extension phase. *CI* confidence interval

**Fig. 2:**
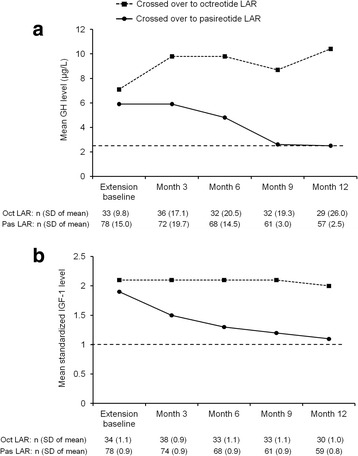
Mean **a** GH and **b** standardized IGF-1 levels at extension baseline and after crossover. The n numbers refer to the number of patients with available data at each month. Oct, octreotide; Pas, pasireotide; SD, standard deviation

Twelve months after crossover to pasireotide LAR, mean GH decreased to 2.5 μg/L (mean decrease of 23.7 %), and mean IGF-1 decreased to 1.1×ULN (mean decrease of 39.9 %) (Fig. 2). Twelve months after crossover to octreotide LAR, mean GH increased to 10.4 μg/L (mean increase of 74.5 %), and mean IGF-1 remained similar to baseline levels (mean increase of 15.9 %).

#### Evaluation of tumor response

**Fig. 3:**
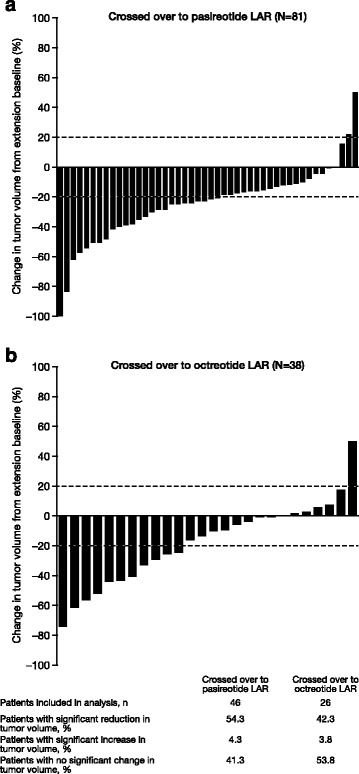
Percentage change from extension baseline in tumor volume 12 months after crossover. Percentage change in tumor volume after **a** switching to pasireotide LAR and **b** switching to octreotide LAR

In the core study, tumor volume decreased from baseline by a mean (±SD) of 40 % (±22) and 38 % (±25) in the pasireotide LAR and octreotide LAR groups, respectively, and 81 and 77 % achieved significant (≥20 %) tumor volume reduction. These results have been described elsewhere [14]. Among evaluable patients who entered the extension and crossed over to opposite treatment, mean (±SD) tumor volume reduction from baseline to month 12 during the core study (prior to switching therapy) was 37 % (±21) in those randomized to pasireotide LAR (*n* = 37) and 34 % (±23) in those randomized to octreotide LAR (*n* = 69). After crossing over to the opposite treatment arm, tumor volume decreased further during the 12 months of the extension study by a mean (±SD) of 25 % (±25) and 18 % (±28) with pasireotide LAR (*n* = 46) and octreotide LAR (*n* = 26), respectively. Significant tumor volume reduction from extension baseline to month 12 after crossover was achieved by 54.3 % (25/46) of pasireotide LAR patients and 42.3 % (11/26) of octreotide LAR patients. Two patients in the pasireotide LAR group and one patient in the octreotide LAR group had a ≥20 % increase in tumor volume 12 months after crossover (Fig. 3).

#### Effect on HRQoL and signs and symptoms

Mean [±SD] AcroQoL scores at extension baseline and 12 months after crossover remained similar in patients who switched to pasireotide LAR (58.9 [±23.1] and 60.3 [±24.3]) and octreotide LAR (59.8 [±22.4] and 61.2 [±21.6]).

The severity of individual symptoms at core baseline was similar for both treatment arms (range of mean values: 0.7–1.2 points in the pasireotide LAR group and 0.8–1.4 points in the octreotide LAR group). The five assessed symptoms of acromegaly remained stable in both groups, although slight improvements were seen in the pasireotide LAR arm. Mean (±SD) changes from extension baseline to 12 months after crossover in the pasireotide LAR and octreotide LAR arms were: headache −0.3 (±0.8) and 0.3 (±0.9); fatigue −0.1 (±0.9) and 0.0 (±0.9); perspiration 0.0 (±0.7) and 0.0 (±0.8); paresthesia −0.1 (±0.9) and 0.1 (±0.8); osteoarthralgia −0.1 (±0.6) and 0.1 (±0.6).

### Safety and tolerability

The safety profiles of both agents were similar to those observed for the corresponding treatment in the core study, that is, the safety profile of pasireotide LAR was similar to that of octreotide LAR, except for the frequency and degree of hyperglycemia.

The most common AEs (pasireotide LAR and octreotide LAR) regardless of a suspected study-drug relationship were hyperglycemia (27.2 and 13.2 %), diarrhea (22.2 and 18.4 %), cholelithiasis (18.5 and 15.8 %), headache (19.8 and 13.2 %), diabetes mellitus (18.5 and 7.9 %), nasopharyngitis (14.8 and 18.4 %), and increased blood creatine phosphokinase (7.4 and 15.8 %) (Table 3). Six (7.4 %) patients in the pasireotide LAR group and six (15.8 %) in the octreotide LAR group experienced serious AEs, of which two and one, respectively, were considered drug related.

**Table 3:** Adverse events regardless of study-drug relationship in ≥10 % of patients in either treatment group. Adverse events are reported from extension baseline up to month 26

	Crossed over to pasireotide LAR (*N* = 81)		Crossed over to octreotide LAR (*N* = 38)	
Adverse event	All grades n (%)	Grade 3/4 n (%)	All grades n (%)	Grade 3/4 n (%)
Total	75 (92.6)	19 (23.5)	34 (89.5)	8 (21.1)
Hyperglycemia	22 (27.2)	4 (4.9)	5 (13.2)	0
Diarrhea	18 (22.2)	0	7 (18.4)	1 (2.6)
Cholelithiasis	15 (18.5)	1 (1.2)	6 (15.8)	1 (2.6)
Headache	16 (19.8)	0	5 (13.2)	0
Diabetes mellitus	15 (18.5)	1 (1.2)	3 (7.9)	0
Nasopharyngitis	12 (14.8)	0	7 (18.4)	0
Arthralgia	10 (12.3)	0	2 (5.3)	0
Increased blood creatine phosphokinase	6 (7.4)	0	6 (15.8)	0
Dizziness	5 (6.2)	0	5 (13.2)	0
Increased blood triglycerides	1 (1.2)	0	4 (10.5)	1 (2.6)

AEs are reported according to terms used by the investigator. AEs are shown in descending order of frequency for patients who crossed over to pasireotide LAR

When common AE terms were pooled, for example, all terms relating to elevations in blood glucose, the most frequently experienced AEs were hyperglycemia related (64.2 and 21.1 %), gallbladder and biliary related (25.9 and 21.1 %), and diarrhea related (22.2 and 18.4 %). Grade 3 or 4 hyperglycemia-related AEs were reported by 12.4 % of patients in the pasireotide LAR group, compared with none in the octreotide LAR group. Eleven (13.6 %) patients discontinued pasireotide LAR because of AEs (all of these patients discontinued because of hyperglycemia-related AEs); no patients discontinued octreotide LAR for this reason. These included five cases of diabetes mellitus or type 2 diabetes mellitus and one case of diabetic ketoacidosis.

Use of concomitant medication was similar between the treatment groups, except for antidiabetic medication. Of the 59 patients who did not have antidiabetic treatment at extension baseline, 13 patients initiated antidiabetic medication after switching to pasireotide LAR. Of these, four, seven and two patients required one, two or at least three antidiabetic medications, respectively; the most commonly administered antidiabetic drugs (≥3 patients) in these patients after crossover were metformin (*n* = 8), dipeptidyl peptidase 4 (DPP-4) inhibitors (*n* = 3), and metformin + DPP-4 inhibitor (*n* = 3) [patients could be counted more than once]. One patient initiated antidiabetic medication with metformin after switching to octreotide LAR.

Mean fasting plasma glucose and glycated hemoglobin (HbA_1c_) levels increased soon after switching from octreotide LAR to pasireotide LAR; mean glucose and HbA_1c_ levels were 104 mg/dL and 6.19 % at extension baseline, 130 mg/dL and 7.03 % at month 3, and 125 mg/dL and 6.68 % at month 12. By contrast, fasting plasma glucose and HbA_1c_ levels decreased to near normal levels within 3 months after switching from pasireotide LAR to octreotide LAR; mean glucose and HbA_1c_ levels were 127 mg/dL and 6.71 % at extension baseline, 104 mg/dL and 6.12 % at month 3, and 103 mg/dL and 5.98 % at month 12.

## Discussion

The results of this crossover extension period of a large Phase III study showed that for patients who had inadequate biochemical control after 12 months of treatment with octreotide LAR, 17.3 % (95 % CI: 9.8, 27.3) achieved biochemical control 12 months after switching treatment to pasireotide LAR. None of the patients who switched from pasireotide LAR to octreotide LAR achieved biochemical control 12 months after crossover. These results suggest that pasireotide LAR may be a promising therapeutic alternative in patients uncontrolled with octreotide LAR.

It is important to note that this extension phase was not designed to detect statistically significant differences between treatment groups. The crossover phase was undertaken to provide additional information on the efficacy and safety of pasireotide LAR and to provide insights into whether non-responders to octreotide LAR or pasireotide LAR may benefit from switching to the opposite therapy. The results reported here support the findings from a recent 24-week, randomized, Phase III study (PAOLA; NCT01137682), which was specifically designed to evaluate the response to pasireotide LAR in patients with acromegaly who were inadequately controlled despite ≥6 months’ treatment with either octreotide LAR or lanreotide Autogel. In this study, a significantly higher proportion of patients achieved biochemical control with pasireotide LAR versus continued treatment with octreotide LAR or lanreotide Autogel at week 24 (15.4 %/20.0 % versus 0 %; *P* = 0.0006 and *P* < 0.0001, respectively) [19]. Given the rarity of acromegaly, few prospective trials have investigated the efficacy and safety of medical therapies in a large number of patients with acromegaly. The results from this crossover phase (*N* = 119) of a large Phase III trial provide a valuable contribution to the existing literature.

In this Phase III study, biochemical control of acromegaly was defined according to targets recommended by clinical consensus guidelines at the time of study initiation (GH <2.5 μg/L and normal IGF-1) [20]. More recently, clinical practice guidelines have advocated reducing GH levels to <1.0 μg/L (using a modern ultrasensitive assay) in the presence of normal IGF-1 levels [13]. It is, therefore, possible that some patients who were considered to be biochemically controlled in this study may not have achieved the more stringent criteria for biochemical control of acromegaly.

Pasireotide LAR is significantly (*P* = 0.007) superior to octreotide LAR at providing GH <2.5 μg/L and normal IGF-1 in patients who have not previously received medical treatment for acromegaly, as shown in the core study. The larger proportion of patients with biochemical control in the pasireotide LAR arm was driven by the higher rate of IGF-1 normalization seen with pasireotide LAR (38.6 % versus 23.6 %; *P* = 0.002) [14]. A similar finding was seen 12 months after switching treatments, with 27.2 % of pasireotide LAR patients and 5.3 % of octreotide LAR patients achieving normal IGF-1. This difference may be due to the broader SSTR binding profile of pasireotide versus octreotide LAR, coupled with the potential role of SSTRs in the disruption of GH signaling on hepatic cells and, subsequently, IGF-1 transcription [21]. In rodents, activation of hepatic SSTR2 and SSTR3 has been shown to downregulate IGF-1 transcription by inactivation of GH-induced signal transduction [21]. As pasireotide would bind with high affinity to both SSTR2 and SSTR3 in the liver, the superiority of pasireotide in normalizing IGF-1 levels may be explained by this proposed mechanism, although studies are required to substantiate this suggestion. Receptor subtype profiling to determine the relative expression of each SSTR was not performed in the current study.

Interestingly, of the 81 patients who switched from octreotide LAR to pasireotide LAR, 34 had GH <2.5 μg/L (but not IGF-1 normalization) at month 12 of octreotide LAR therapy, and 36 had GH <2.5 μg/L (with or without IGF-1 normalization) 12 months after switching to pasireotide LAR therapy. This finding is consistent with the core study result that the two drugs have a similar effect on GH, but that pasireotide LAR has a greater effect on reducing IGF-1 levels. The similar SSTR2 binding affinity of pasireotide and octreotide may be the mechanism underlying these parallel GH findings and supports the previously established finding that IGF-1 is the driver of the superior efficacy of pasireotide LAR.

A few patients entered the crossover phase before a protocol amendment was implemented; these patients received open-label pasireotide LAR (*n* = 19) during the crossover phase. As a result, there is a possibility of bias in favor of pasireotide LAR for this small subset of patients. Patients who completed the core study before the amendment could not receive octreotide LAR in the extension phase, either as continued therapy or by switching therapy; this feature may have contributed to the imbalance in crossover cohort numbers. Patient records were reviewed retrospectively and it was estimated that 14 additional patients may have been eligible to cross over to octreotide LAR if the protocol revision had been made earlier. It is unknown if these patients would have entered the crossover extension and/or achieved an improved response.

The crossover phase of this large Phase III trial provided the opportunity to examine the safety profile of pasireotide LAR and octreotide LAR after switching medical therapies. After crossover, the safety profiles of pasireotide LAR and octreotide LAR were similar to those seen in the core study; pasireotide LAR has a similar safety profile to that of octreotide LAR, except for a greater frequency and degree of hyperglycemia-related AEs. This study suggests that the hyperglycemia associated with pasireotide LAR is reversible, as fasting plasma glucose and HbA_1c_ levels declined rapidly in patients who were switched from pasireotide LAR to octreotide LAR. However, further investigation is needed to confirm this. It has recently been shown in healthy volunteers that while pasireotide inhibits insulin secretion and incretin response and suppresses glucagon levels to a more modest degree, it does not affect insulin sensitivity [22]. Patients receiving pasireotide treatment should be closely monitored for changes in glucose homeostasis, with antidiabetic medication initiated promptly if hyperglycemia occurs. Based on the results of mechanistic studies in healthy volunteers, a panel of experts have recently recommended that hyperglycemia associated with pasireotide in patients with Cushing’s disease should be managed by the following staged intensification of antidiabetic treatment until glycemic control is achieved: (i) initiation of metformin; (ii) addition of a DPP-4 inhibitor; (iii) switch from the DPP-4 inhibitor to a glucagon-like peptide 1 receptor agonist; (iv) initiation of insulin therapy [23]. Given the relationship between pasireotide and hyperglycemia, these recommendations may also be applicable to patients with acromegaly.

Pegvisomant, a GH receptor antagonist, is currently used in patients who have an inadequate response to maximal doses of somatostatin analogues [24]. Initial results of pegvisomant in patients with acromegaly (*n* = 160) showed that normal IGF-1 levels were achieved in 87 of the 90 (97 %) patients who received pegvisomant for at least 12 months [25]. Results from the ACROSTUDY registry have demonstrated that pegvisomant, as either mono- or combination therapy, provides IGF-1 normalization in ~63 % of patients after 5 years of treatment in clinical practice [26]. However, in a recent study, combined treatment with pegvisomant and long-acting somatostatin analogues normalized IGF-1 levels in 97 % of patients who were uncontrolled with somatostatin analogues alone [27]. Interestingly, in addition to its IGF-1-lowering effect, pegvisomant may also improve glucose homeostasis in patients with diabetes mellitus [28]. However, pegvisomant does not target the pituitary tumor and has no effect on tumor volume. In the current study, tumor volume decreased further during the 12 months of the extension study in both groups, with significant tumor volume reduction demonstrated in nearly half of all patients who participated in the extension.

## Conclusions

The results of this study, which support those from the recent PAOLA study, suggest that pasireotide LAR may be an effective treatment option in patients with acromegaly who are inadequately controlled with first-generation somatostatin analogues.

### Availability of data and materials

Not applicable.

## Additional file

Additional file 1: Appendix. (DOCX 31.6 kb)

## Abbreviations

AE: adverse event
CI: confidence interval
CTCAE: common terminology criteria for adverse events
DPP-4: dipeptidyl peptidase 4
GH: growth hormone
HbA_1c_: glycated hemoglobin
HRQoL: health-related quality of life
IGF-1: insulin-like growth factor 1
LAR: long-acting release
SD: standard deviation
SSTR: somatostatin receptor
ULN: upper limit of normal
