# Supplementary material for: Switching patients with acromegaly from octreotide to pasireotide improves biochemical control: crossover extension to a randomized, double-blind, Phase III study
Source: BMC Endocr Disord. 2016 Apr 2;16:16. doi: 10.1186/s12902-016-0096-8 (PMC4818908; doi:10.1186/s12902-016-0096-8)
Supplement: Additional file 1: — Appendix. (DOCX 31.6 kb) [file 12902_2016_96_MOESM1_ESM.docx]

**Supplementary Appendix**

**Switching patients with acromegaly from octreotide to pasireotide improves biochemical control: crossover extension to a randomized, double-blind,
Phase III study**

**Table of contents**

Tumor volume assessment 2

GH and IGF-1 measurement and assays 2

Pasireotide C2305 Study Group 3

Independent ethics committees and institutional review boards 4

**Tumor volume assessment**

The intra-observer variability of the blinded central reader has been assessed independently by a third-party organization with experience in radiological measurement, repeatability and reproducibility. The central reader measured pituitary tumor volumes in patients undergoing treatment, then re-read images of 10 randomly selected patients from 29 time points in total, in a blinded manner, and not earlier than 3 weeks after the original reading. Variability analysis was performed on a time-point-by-time-point and case basis. For the time-point-by-time-point analysis, the absolute and percentage differences between the volumes rendered at the first and secondary reviews were determined at each time point. For the case analysis, the volume percentage change at each post-baseline time point in comparison with baseline was calculated for the first and secondary reviews and compared against each other. The results of the analysis were consistent, reproducible and, in most cases, unaffected by complex tumor anatomy, small absolute tumor size and/or poor image quality. The results of the case analysis showed percentage change differences in excess of 10% in 26% of the cases, reflecting a reasonable level of reading consistency. The variability in central reader measurements falls well within the 20% volume change threshold specified in the study as indicating a significant change in tumor volume.

**GH and IGF-1 measurement and assays**

Serum GH and IGF-1 were measured using validated chemiluminescent immunometric assays (Immulite^®^ 2000/1000; Diagnostic Products Corp [Siemens], Los Angeles, CA, USA; GH International Reference Preparation [IRP] WHO NIBSC 2nd IS 98/574; IGF-1 IRP WHO NIBSC 1st IRR 87/518). The lower limit of detection for GH was 0.1μg/L, with intra- and inter-assay coefficients of variation ≤6.6%. For IGF-1, the lower limit of detection was 20μg/L, with intra- and inter-assay coefficients of variation ≤6.7%. IGF-1 values were compared with age- and sex-standardized normal values. All samples except for those from China were analyzed using the Immulite^®^ 2000 assay by Quest Diagnostics Nichols Institute Laboratory, San Juan Capistrano, CA, USA between March 2008 and March 2010, then by Quest Diagnostics Clinical Trials Laboratory, Valencia, CA, USA from March 2010 onwards. Samples from China were analyzed using the Immulite^®^ 1000 assay by Kingmed Diagnostics, Guangzhou, China. Subsequent to receiving a notification from Siemens regarding the Immulite^®^ IGF-1 assay, Quest Diagnostics reviewed all quality control data collected from the three laboratories and verified that all IGF-1 data are valid.

**Pasireotide C2305 Study Group**

Members of the Pasireotide C2305 Study Group include: G Akcay, A Arafat, S Arellano, A Barkan, J Bertherat, M Bex, C Boguszewski, J Bollerslev, F Borson-Chazot,
M Bronstein, T Brue, O Bruno, F Casanueva, C-N Chang, T-C Chang, P Chanson,
A Chervin, C Chik, A Colao, B Corvilain, L De Marinis, E Degli Uberti, L Duan,
B Edén Engstrom, T Erbas, AJ Farrall, M Faria, M Fleseriu, P Freda, M Gadelha,
E Ghigo, B Glaser, M Gordon, E Grineva, F Gu, M Guitelman, A Heaney, G Houde,
L Katznelson, Y Khalimov, K-W Kim, M-S Kim, S-W Kim, P Laurberg, E-J Lee,
W Ludlam, J Marek, E Martino, M McPhaul, M Mercado, F Minuto, R Montenegro,
L Naves, G Ning, G Piaditis, A Pico Alfonso, V Pronin, S Quinn, K Racz, W Rojas,
L Rozhinskaya, R Salvatori, S Samson, D Sandeman, J Schopohl, O Serri, C-C Shen,
I Shimon, J Soler Ramon, A Tabarin, G T’Sjoen, N Unger, AJ van der Lely, E Venegas Moreno, S Waguespack, and W Zgliczyński.

**Independent ethics committees and institutional review boards**

***Argentina***

Comité de Docencia e Investigación del Centro de Estudios Metabólicos y Endócrinos, Buenos Aires Capital City, Buenos Aires, Argentina

Comité de Docencia e Investigación Hospital Santa Lucía, Buenos Aires Capital City, Buenos Aires, Argentina

Comité de Revisión Institucional del Instituto Mé dico Especializado (IME), Buenos Aires Capital City, Buenos Aires, Argentina

***Belgium***

Ethics Committee, UZ Gent, Gent, Belgium

Ethics Committee, UZ Gasthuisberg, Leuven, Belgium

Ethics Committee, Hopital Erasme, Bruxelles, Belgium

***Brazil***

Comitê de Ética em Pesquisa da Faculdade de Medicina da Universidade de São Paulo, Sao Paulo, Brazil

Comitê de Ética em Pesquisa em Seres Humanos do Hospital de Clínicas da Universidade Federal do Paraná (HCUFPR), Curitiba, Brazil

Comitê de Ética em Pesquisa em Seres Humanos do Hospital Universitário Clementino Fraga Filho, Rio de Janeiro, Brazil

Comitê de Ética em Pesquisa do Hospital Universitário da Universidade Federal Do Maranhao, São Luís, Brazil

Comitê de Ética em Pesquisa em Seres Humanos do Hospital Univesitário Walter Cantí dio/Universidade Federal do Ceará, Fortaleza, Brazil

Comitê de Ética em Pesquisa da Faculdade de Medicina da Universidade de Brasília (FM/UNB), Brasília, Brazil

***Canada***

Centre de recherché, Comités d'évaluation scientifique et d'éthique de la recherche, Montréal, Québec, Canada

Health Research Ethics Board – University of Alberta, Alberta, Ontario, Canada

Institutional Review Board Services, Aurora, Ontario, Canada

Capital Health Research Ethics Board, Halifax, Nova Scotia, Canada

Comité d'éthique de la recerche en santö chez l’humain, CHU de Sherbrooke Hôpital Fleurimont, Fleurimont, Québec, Canada

***China***

IEC OF Ruijin Hospital Shanghai Jiao Tong University School of Medicine, Shanghai, China

IEC OF Peking Union Medical College Hospital, Beijing, China

***Colombia***

Comité de Ética en Investigación con Seres Humanos HSJ-FUCS (CEISH), Bogotá, Colombia

***Czech Republic***

Eticka komise pri VFN, Prague, Czech Republic

***Denmark***

Den Videnskabsetiske komite for region Nordjylland, Ålborg Øst, Denmark

***France***

Comité de Protection des Personnes Sud–Est IV, Lyon Centre Régional de Lutte contre le cancer Léon Bérard, Lyon, France

***Germany***

Ethik-Kommission der Medizinischen Fakultät der Universität Duisburg-Essen, Essen, Germany

Landesamt für Gesundheit und Soziales Ethik-Kommission des Landes Berlin, Berlin,
Germany

Landesamt für Gesundheit und Soziales Berlin Geschäftsstelle der Ethik-Kommission des Landes Berlin, Berlin, Germany

Ethik-Kommission der Medizinischen Fakultät der Ludwig-Maximilians-Universität München, München, Germany

***Greece***

Scientific Council, General Hospital of Athens ‘G. Gennimatas’, Athens, Greece

***Hungary***

Medical Research Council Ethics Committee for Clinical Pharmacology, Budapest, Hungary

***Israel***

Hadassah Ein Kerem, Hebrew University MC, Jerusalem, Israel

Rabin Medical Center, Petah-Tikva, Israel

***Italy***

Comitato Etico della Provincia di Ferrara, Ferrara, Italy

Comitato Etico per le Attivita Biomediche Carlo Romano dell ‘Universitat’ degli Studi, Napoli, Italy

Comitato Etico dell’Azienda Ospedaliera Universitaria Pisana, Pisa, Italy

Comitato Etico Dell’azienda Azienda Ospedaliera Universitaria s Giovanni Battista di Torino, Torino, Italy

Comitato Etico dell’Azienda Ospedaliera Universitaria S Martino di Genova, Genova, Italy

Comitato Etico Per La Sperimentazione Dell’azienda Ospedaliera Di Padova, Padova, Italy

Comitato Etico Dell’universita’ Cattolica Del Sacro Cuore, Policlinico Gemelli, Rome, Italy

**Korea, Republic of**

Asan Medical Center IRB (AMC IRB), Seoul, Republic of Korea

Samsung Medical Center IRB, Seoul, Republic of Korea

Severance Hospital IRB, Seoul, Republic of Korea

KyungHee University Medical Center IRB, Seoul, Republic of Korea

***Mexico***

Comite de Etica del IMSS, Mexico City, Mexico

***Netherlands***

CMO Arnhem Nijmegen, Nijmegen, Netherlands

METC Erasmus MC, Rotterdam, Netherlands

***Norway***

Regional komité for medisinsk og helsefaglig forskningsetikk Sør-Øst C, Oslo, Norway

***Poland***

Komisja Bioetyczna Centrum Medyczne Kształcenia Podyplomowego w Warszawie, Warszawa, Poland

***Russia***

Local Ethics Committee of the Federal State Institution ‘Endocrinology Research Center’ of Rosmedtekhnologiy, Moscow, Russia

Inter-College Ethics Committee under the Association of Medical and Pharmaceutical Colleges, Moscow, Russia

Ethics Committee of SM Kirov Medical Military Academy, Saint Petersburg, Russia

Ethics Committee of Federal Almazov Heart, Blood and Endocrinology Centre, Saint Petersburg, Russia

***Spain***

CEIC Hospital General Universitario de Alicante, Alicante, Spain

CEIC Hospital Universitario Virgen Macarena, Sevilla, Spain

CEIC de Galicia, Santiago de Compostela, A Coruña, Spain

CEIC Hospital Universitari de Bellvitge, Hospitalet de Llobregat, Barcelona, Spain

***Sweden***

Etikprövningsnämnden i Lund, Lund, Sweden

***Switzerland***

Ethikkommission des Kantons St Gallen, Kantonsspital St Gallen, St Gallen, Switzerland

***Taiwan***

National Taiwan University Hospital – Research Ethics Committee, Taipei, Taiwan

Taichung Veterans General Hospital – The Institutional Review Board, Taichung, Taiwan

Chang-Gung Medical Foundation – Institutional Review Board, Taoyuan, Taiwan

***Turkey***

Hacettepe Üniversitesi, Sıhhiye/Ankara, Turkey

Atatürk Üniversitesi Tıp Fakültesi, İlaç Araştı rmaları yerel Etik kurulu, Erzurum, Turkey

***United Kingdom***

Sunderland Research Ethics Committee, Jarrow, United Kingdom

***United States***

Administration Panel on Human Subjects, Stanford, CA, USA

Western Institutional Review Board, Olympia, WA, USA

UCLA Office of Protection of Research Subjects, Los Angeles, CA, USA

Oregon Health & Science University Institutional Review Board, Portland, OR, USA

The University of Texas MD Anderson Cancer Center Surveillance Committee, Houston, TX, USA

University of Michigan Institutional Review Board (IRBMED), Ann Arbor, MI, USA

University of Texas Southwestern Medical Center, Dallas, TX, USA

Johns Hopkins Medicine Institutional Review Board Reed Hall, Baltimore, MD, USA

Institutional Review Board for Human Subject Research for Baylor College of Medicine and Affiliated Hospitals, Houston, TX, USA
